# Evaluating the Accuracy of Gemini 2.0 Advanced and ChatGPT 4o in Cataract Knowledge: A Performance Analysis Using Brazilian Council of Ophthalmology Board Exam Questions

**DOI:** 10.7759/cureus.79565

**Published:** 2025-02-24

**Authors:** Diego Casagrande, Mauro Gobira

**Affiliations:** 1 Ophthalmology, Vision institute (IPEPO), São Paulo, BRA

**Keywords:** artificial intelligence, board exam, cataract, chatgpt, gemini, large language models, ophthalmology

## Abstract

Introduction: Large language models (LLMs) like Gemini 2.0 Advanced and ChatGPT-4o are increasingly applied in medical contexts. This study assesses their accuracy in answering cataract-related questions from Brazilian ophthalmology board exams, evaluating their potential for clinical decision support.

Methods: A retrospective analysis was conducted using 221 multiple-choice questions. Responses from both LLMs were evaluated by two independent ophthalmologists against the official answer key. Accuracy rates and inter-evaluator agreement (Cohen’s kappa) were analyzed.

Results: Gemini 2.0 Advanced achieved 85.45% and 80.91% accuracy, while ChatGPT-4o scored 80.00% and 84.09%. Inter-evaluator agreement was moderate (κ = 0.514 and 0.431, respectively). Performance varied across exam years.

Conclusion: Both models demonstrated high accuracy in cataract-related board exam questions, supporting their potential as educational tools. However, moderate agreement and performance variability indicate the need for further refinement and validation.

## Introduction

Artificial intelligence (AI) has made significant strides in medicine, particularly with the development of large language models (LLMs) [1]. These deep neural network-based models are trained on vast text datasets, enabling them to generate contextually relevant responses and assist in various applications [1,2]. In healthcare, LLMs such as ChatGPT (OpenAI, San Francisco, CA) and Gemini (Google AI, Mountain View, CA) have demonstrated potential in clinical decision support, medical education, and diagnostic assistance [1-4].

Ophthalmology is a medical discipline that relies on a comprehensive understanding of ocular diseases, including cataracts, a leading cause of visual impairment worldwide [5]. Cataracts result from the progressive opacification of the eye’s natural lens, leading to visual decline and, if untreated, blindness [5]. In Brazil, the Brazilian Council of Ophthalmology (BCO) plays a central role in certifying ophthalmologists through rigorous annual board examinations [6]. Among these assessments, the Theoretical Exam II evaluates core clinical ophthalmology topics, including cataract diagnosis and management [6]. Given the importance of these exams in standardizing ophthalmology training, the ability of AI models to accurately respond to board exam questions warrants investigation.

This study aims to assess the accuracy of two state-of-the-art LLMs, Gemini 2.0 Advanced and ChatGPT-4o, in answering cataract-related questions from BCO exams (2006-2024). Given the lack of studies evaluating these latest models in this specific context, this research addresses a critical gap in the literature. Additionally, the inter-evaluator agreement is analyzed to assess response consistency. Understanding the strengths and limitations of these models is essential for determining their potential role in clinical decision-making in ophthalmology.

## Materials and methods

Study design and ethical considerations

This study was designed as a retrospective, cross-sectional analysis to evaluate the accuracy of two LLMs, Gemini 2.0 Advanced (Google LLC, Mountain View, California, United States) and ChatGPT-4o (OpenAI, San Francisco, California, United States), in answering cataract-related questions from the BCO board examinations. Both models were tested under identical conditions, with responses compared against the official answer key provided by the BCO, serving as the reference standard. To assess the reproducibility of AI-generated responses, two independent board-certified ophthalmologists evaluated the model outputs. In this context, reproducibility refers to the consistency of AI-generated answers when presented with the same questions under the same conditions.

Since this study involved the analysis of publicly available examination data and did not include human subjects, formal ethical approval was not required. However, the study adhered to principles of research integrity, transparency, and data accuracy throughout the evaluation process. This research aims to contribute to the ongoing discourse on the role of AI-driven LLMs in ophthalmology education and decision-making.

Question selection and data source

The dataset comprised only text-based multiple-choice questions on cataracts from the Theoretical Exam II of the BCO, excluding any questions containing images or videos. Covering exams administered between 2006 and 2024, this examination focuses on clinical ophthalmology and excludes basic science subjects such as anatomy, histology, and embryology. The analysis included questions on cataract pathophysiology, diagnosis, medical and surgical management, and post-operative complications. Questions annulled or deemed invalid by the BCO were excluded from the study. Each question was assigned a unique identifier for documentation and tracking. All selected questions were in Portuguese, and the evaluation period spanned from January 20 to January 30, 2025. To ensure data integrity and validity, all questions were sourced from official BCO archives.

Large language model (LLM) response generation and evaluation

Each question was input verbatim into both Gemini 2.0 Advanced and ChatGPT-4o. The most recent versions of each model available at the time of data collection were used. No additional context, modifications, or supplementary instructions were provided to the models to ensure an unbiased evaluation of their baseline reasoning capabilities. AI responses were not pre-processed or reformatted before evaluation to ensure an objective comparison with the official answer key. Each model was prompted once per question, and only the first response was considered for analysis to reflect real-world usage.

The AI-generated responses were independently evaluated by two board-certified ophthalmologists, each with over five years of clinical experience in cataract management. The evaluators were blinded to the source of the responses (i.e., whether they originated from ChatGPT-4o or Gemini 2.0) to minimize examiner bias and did not have access to each other's assessments. Each response was classified as either Correct or Incorrect based on its alignment with the official BCO answer key. Responses that contained partially correct information but did not fully align with the official answer key were classified as Incorrect.

Accuracy assessment and statistical analysis

The primary outcome was the accuracy rate of each LLM, measured as the percentage of correctly answered questions. Performance trends over the years were assessed, and descriptive statistics, including the mean accuracy, were calculated for each model. Inter-evaluator agreement was assessed using Cohen’s kappa (κ), interpreted as follows: 0.00-0.20 (slight), 0.21-0.40 (fair), 0.41-0.60 (moderate), 0.61-0.80 (substantial), and 0.81-1.00 (almost perfect). Statistical analyses were performed using IBM SPSS Statistics (IBM Corp., Armonk, NY) and R software (R Foundation for Statistical Computing, Vienna, Austria), with significance set at p<0.05.

## Results

A total of 221 cataract-related multiple-choice questions were identified from the BCO board examinations conducted between 2006 and 2024. After excluding one nullified question, a final dataset of 220 valid questions was included in the analysis. The number of cataract-related questions per year ranged from five to 13, with the highest frequency recorded in 2008 (13 questions) and the lowest in 2018 (five questions). The average number of cataract-related questions per year was 9.73.

**Figure 1:**
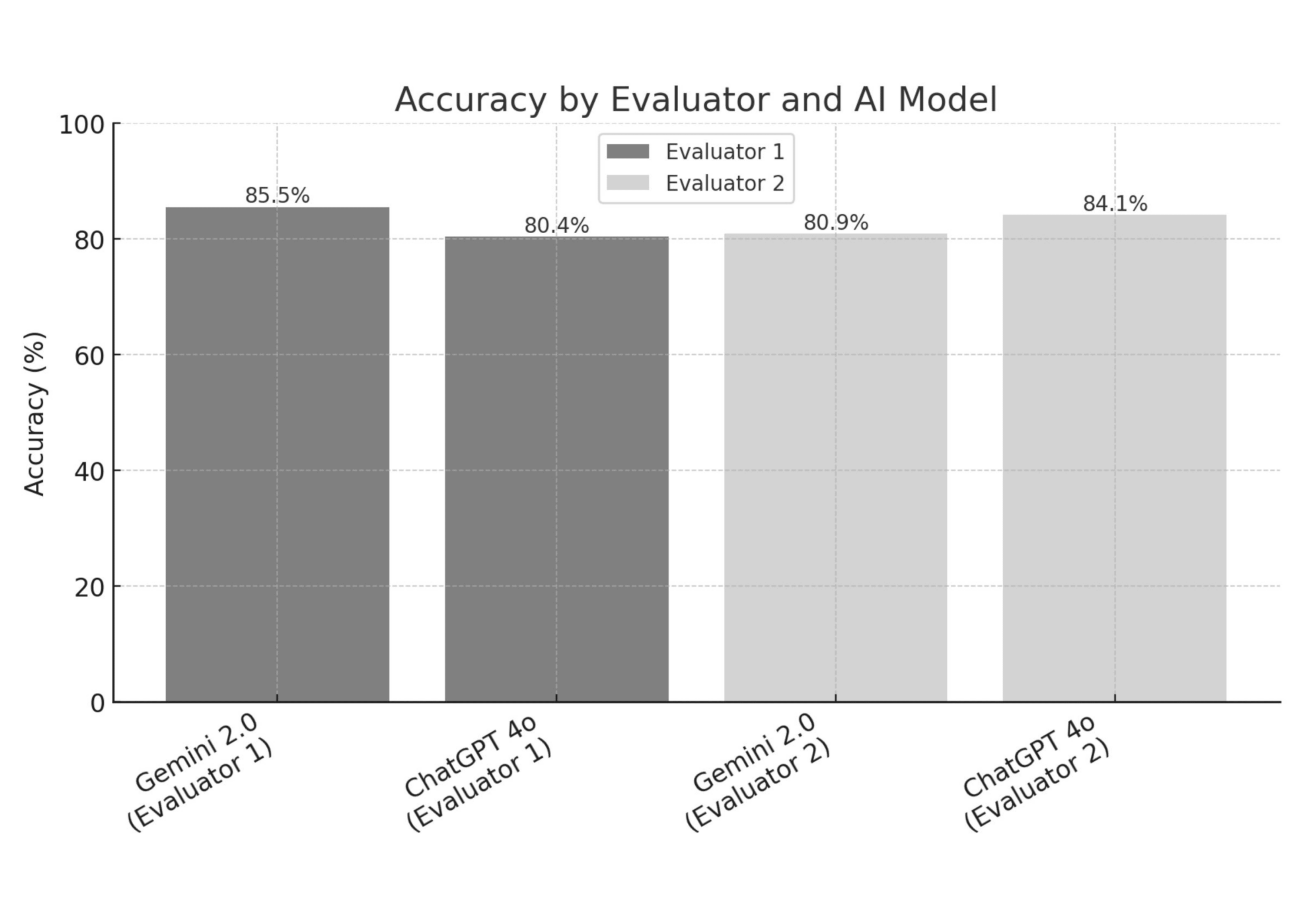
Accuracy by the evaluator and AI model The image was created by the authors of this article.

The accuracy of Gemini 2.0 Advanced and ChatGPT-4o was assessed by two independent evaluators. For Gemini 2.0, Evaluator 1 reported an accuracy of 85.45% (95% confidence interval [CI]: 80.76%-90.15%), while Evaluator 2 recorded a slightly lower accuracy of 80.91% (95% CI: 75.67%-86.14%). In contrast, ChatGPT-4o achieved an accuracy of 80.00% (95% CI: 74.67%-85.33%) as evaluated by Evaluator 1, whereas Evaluator 2 observed a higher accuracy of 84.09% (95% CI: 79.22%-88.96%). These results indicate that both LLMs performed comparably well, with accuracy levels exceeding 80% across evaluations (Figure 1).

When analyzed across different exam years, Gemini 2.0 achieved 100% accuracy in 2008, as assessed by Evaluator 1, but its accuracy declined to 71.43% in 2010, indicating year-to-year variability (Table 1). Similarly, ChatGPT-4o exhibited fluctuations in performance, with its highest accuracy reaching 100% in 2007 (Evaluator 1), while its lowest recorded accuracy occurred in 2008 (61.54%).

**Table 1:** Accuracy comparison of AI models by evaluator and year This table presents the performance of Gemini 2.0 Advanced and ChatGPT-4o in answering cataract-related board examination questions from the Brazilian Council of Ophthalmology (BCO) over multiple years. Accuracy is reported as both the percentage of correct responses and the absolute number of correct answers out of the total questions for each year.

Year	Gemini 2.0 - Evaluator 1 (Correct/Total, %)	ChatGPT-4o - Evaluator 1 (Correct/Total, %)	Gemini 2.0 - Evaluator 2 (Correct/Total, %)	ChatGPT-4o - Evaluator 2 (Correct/Total, %)
2006	10/12 (83.3%)	10/12 (83.3%)	10/12 (83.3%)	10/12 (83.3%)
2007	10/12 (83.3%)	12/12 (100.0%)	10/12 (83.3%)	11/12 (91.7%)
2008	13/13 (100.0%)	8/13 (61.5%)	12/13 (92.3%)	12/13 (92.3%)
2009	11/13 (84.6%)	12/13 (92.3%)	11/13 (84.6%)	12/13 (92.3%)
2010	10/14 (71.4%)	10/14 (71.4%)	11/14 (78.6%)	13/14 (92.9%)
2011	10/11 (90.9%)	7/11 (63.6%)	7/11 (63.6%)	8/11 (72.7%)
2012	9/11 (81.8%)	9/11 (81.8%)	6/11 (54.5%)	10/11 (90.9%)
2013	11/12 (91.7%)	8/12 (66.7%)	10/12 (83.3%)	9/12 (75.0%)
2014	12/13 (92.3%)	9/13 (69.2%)	13/13 (100.0%)	9/13 (69.2%)
2015	10/10 (100.0%)	9/10 (90.0%)	9/10 (90.0%)	10/10 (100.0%)
2016	11/14 (78.6%)	12/14 (85.7%)	13/14 (92.9%)	12/14 (85.7%)
2017	8/9 (88.9%)	8/9 (88.9%)	7/9 (77.8%)	8/9 (88.9%)
2018	5/7 (71.4%)	5/7 (71.4%)	4/7 (57.1%)	4/7 (57.1%)
2019	10/11 (90.9%)	9/11 (81.8%)	8/11 (72.7%)	9/11 (81.8%)
2020	11/11 (100.0%)	10/11 (90.9%)	11/11 (100.0%)	10/11 (90.9%)
2021	9/11 (81.8%)	9/11 (81.8%)	8/11 (72.7%)	10/11 (90.9%)
2022	9/10 (90.0%)	7/10 (70.0%)	6/10 (60.0%)	8/10 (80.0%)
2023	8/12 (66.7%)	9/12 (75.0%)	8/12 (66.7%)	7/12 (58.3%)
2024	8/11 (72.7%)	10/11 (90.9%)	10/11 (90.9%)	8/11 (72.7%)

To assess consistency between evaluators, Cohen’s kappa (κ) statistic was calculated as a measure of inter-rater reliability. Gemini 2.0 achieved a Cohen’s kappa of 0.514, indicating moderate agreement, while ChatGPT-4o demonstrated a slightly lower agreement, with a κ value of 0.431. These results indicate a moderate level of agreement between evaluators.

Overall, these results demonstrate that both models perform well in cataract-related board exam questions but are limited by moderate inter-evaluator agreement and year-to-year performance variability.

## Discussion

The findings of this study suggest that LLMs can achieve high accuracy in answering cataract-related board examination questions, highlighting their potential as complementary tools in clinical decision support for ophthalmology. Both Gemini 2.0 Advanced and ChatGPT-4o consistently achieved accuracy rates exceeding 80%, although performance varied over the years. These results align with previous research on the use of LLMs in ophthalmology, where advanced models have demonstrated superior performance compared to earlier versions [7-21]. However, despite their promising potential, these findings should be analyzed within the broader context of applying language models in ophthalmology, considering their limitations, possible inaccuracies, and the need for refinement before practical implementation. To the best of our knowledge, this is the first study to directly compare the performance of Gemini 2.0 Advanced and ChatGPT-4o in cataract-specific board exam questions, providing novel insights into their respective capabilities in a structured assessment.

A notable finding was the variability in model performance over the years. While Gemini 2.0 achieved 100% accuracy in 2008, its performance dropped to 71.43% in 2010. ChatGPT-4o showed similar fluctuations, with its highest recorded accuracy in 2007 (100%) and its lowest in 2008 (61.54%). These variations may be associated with differences in question complexity, terminology, or exam structure in certain years. Future studies should investigate whether specific question formats contribute to this inconsistency.

Previous studies have shown that advanced LLMs, such as ChatGPT-4, outperform their predecessors across various medical domains [11,12]. This trend has been observed in AI evaluations of medical licensing exams, including the United States Medical Licensing Examination (USMLE) and the Fellowship of the Royal College of Ophthalmologists (FRCOphth) exams [7-9]. In ophthalmology, ChatGPT-4 has demonstrated higher accuracy when tested on specialty-specific questions. A study evaluating its performance on 467 StatPearls ophthalmology questions reported that GPT-4 achieved an accuracy of 73.2%, significantly outperforming GPT-3.5 (55.5%) and human professionals (58.3%) (p < 0.001) [11]. However, its performance was lower in the “Lens and Cataract” category, suggesting possible topic-specific limitations [11].

The results of this study suggest that Gemini 2.0 and ChatGPT-4o significantly outperformed earlier LLM versions. Previous investigations reported that ChatGPT-3.5 achieved 73.1% accuracy on Brazilian ophthalmology board exam questions related to cataracts, a lower performance compared to the more advanced models in this study [16]. Another study reported even lower accuracy rates for cataract-related questions, with ChatGPT-3.5 reaching 45% and ChatGPT-4 52%, further reinforcing the performance improvements observed with the newer models in this study [11]. The moderate agreement between evaluators (Cohen’s kappa: 0.514 for Gemini 2.0, 0.431 for ChatGPT-4o) may be associated with the lack of reproducibility in the AI-generated responses, leading to variations in structure, coherence, and content. These findings highlight the need to enhance model predictability to improve reliability in medical practice.

Despite their promising performance, LLMs still have limitations. Previous research has identified instances where these models generate incorrect or misleading answers with high confidence, particularly when dealing with complex or ambiguous clinical scenarios [15,16]. Additionally, challenges remain in mathematical reasoning and image-based diagnostics, both of which are crucial for ophthalmological decision-making [16-18]. A major limitation of this study is the exclusion of image-based questions, which are fundamental in ophthalmology assessments. Since cataract diagnosis and management often rely on slit-lamp images, fundus photography, and optical coherence tomography (OCT), restricting the analysis to text-based questions may have overestimated the clinical applicability of AI. Previous studies have already shown that AI models struggle with image-based reasoning, particularly in differentiating subtle pathological findings [16-21]. Moreover, it is not possible to completely rule out the possibility that the models were exposed to exam questions during training. Future studies should investigate whether AI performance may have been influenced by prior access to similar questions.

To overcome these limitations, future research should focus on refining LLMs for ophthalmology applications, incorporating specialized datasets, and developing multimodal AI models capable of processing both text and medical images. Evaluating these models on image-based questions, complex clinical scenarios, and multi-step clinical reasoning tasks will be essential for a more comprehensive assessment of their capabilities. Expanding these studies beyond cataract-related assessments to include other ophthalmic conditions will help clarify their role in medical education and clinical decision-making.

## Conclusions

This study shows that Gemini 2.0 Advanced and ChatGPT-4o achieve over 80% accuracy in cataract-related board exam questions, highlighting their potential as supplementary tools in ophthalmology clinical decision-making and support. However, performance variability and moderate inter-rater agreement indicate a lack of response reproducibility, requiring further refinement. The exclusion of image-based questions also limits the generalizability of these findings. Given these limitations, LLMs can assist in clinical decision-making but should not replace expert judgment. Future research should focus on enhancing response consistency, evaluating AI across more ophthalmic conditions, and integrating multimodal models to improve applicability in ophthalmology.
